# Genetic Profile and Associated Characteristics of 150 Korean Patients with Retinitis Pigmentosa

**DOI:** 10.1155/2021/5067271

**Published:** 2021-10-21

**Authors:** You Na Kim, Yoon Jeon Kim, Chang Ahn Seol, Eul-Ju Seo, Joo Yong Lee, Young Hee Yoon

**Affiliations:** ^1^Department of Ophthalmology, Asan Medical Center, University of Ulsan College of Medicine, Seoul 05505, Republic of Korea; ^2^GC Genome, Gyeonggi-do 16924, Republic of Korea; ^3^Department of Laboratory Medicine, Asan Medical Center, University of Ulsan College of Medicine, Seoul 05505, Republic of Korea

## Abstract

**Purpose:**

Retinitis pigmentosa (RP) shows great diversity between genotypes and phenotypes, and it is important to identify the causative genes. This study aimed to analyze the molecular profiles, associated ocular characteristics, and progression of RP in Korean patients.

**Methods:**

All the genetic variants in patients with RP, identified using targeted next-generation sequencing (NGS) with a panel of 88 RP-related genes between November 2018 and November 2019, were retrospectively reviewed. All the patients underwent comprehensive ophthalmological evaluations, and their clinical and family histories were recorded. The best-corrected visual acuity (BCVA) deterioration and photoreceptor disruption progression rates were determined based on the major causative mutational genes using nonlinear mixed models, and the differences among them were investigated using the interaction effect.

**Results:**

Among the 144 probands, 82 variants in 24 causative genes were identified in 77 families (53.5%). Most of the RP cases were associated with autosomal recessive variants (*N* = 64 (44.4%)), followed by autosomal dominant (*N* = 10 (6.9%)) and X-linked variants (*N* = 3 (2.1%)). The four most frequently affected genes were EYS (*N* = 15 (10.4%)), USH2A (*N* = 12 (8.3%)), PDE6B (*N* = 9 (6.3%)), and RP1 (*N* = 8 (5.6%)). Epiretinal membranes and cystoid macular edema were frequently noted in the patients with USH2A (75.0%) and PDE6B (50.0%) variants, respectively. During the follow-up period, the BCVA and photoreceptor disruption changes were significantly different among the patients carrying the four common causative genes (*P*=0.014 and 0.034, resp.). Patients with PDE6B variants showed faster BCVA changes (0.2 LogMAR/10 years), and those with USH2A variants showed the fastest ellipsoid zone disruptions (−170.4 *µ*m/year).

**Conclusion:**

In conclusion, our genetic analysis using targeted NGS provides information about the prevalence of RP-associated mutations in Korean patients. Delineating clinical characteristics according to genetic variations may help clinicians identify subtype features and predict the clinical course of RP.

## 1. Introduction

Retinitis pigmentosa (RP, MIM#268000), which is the most common genetic retinopathy, is defined as a heterogeneous group of diseases that have various causes and unique mechanisms; however, each disease within this group ultimately results in the deterioration of vision [1]. Most affected patients present with isolated retinal disorders (i.e., nonsyndromic RP). However, approximately 20%–30% of patients present with multiorgan manifestations, such as hearing loss, obesity, and musculoskeletal disorders combined with retinal disorders (i.e., syndromic RP) [2]. Although RP is associated with a range of diverse, yet overlapping symptoms, it primarily affects the rod and cone photoreceptors and is characterized by progressive night blindness, reduced electroretinographic responses, constriction, and gradual loss of the visual field, and a subsequent loss of visual acuity [3].

The molecular etiology of RP is quite complicated, as genetic heterogeneity is found among RP cases. Autosomal recessive, autosomal dominant, and X-linked recessive forms of RP exist [4]. Moreover, approximately 50% of RP cases are sporadic. It has been postulated that mutations in at least 80 genes are responsible for causing RP [5]. Because RP is known to show great diversity between genotypes and phenotypes, the identification of causative genes is critical. The recent introduction of gene panel-based sequencing offers more effective methods for the molecular analysis of disease-causing genes [6]. Several studies have evaluated the nationwide incidence of RP in Korea using population data from the Korean National Health Insurance System or from tertiary clinics. However, these studies were limited to reporting on the incidence, demographic characteristics, and ophthalmic characteristics of patients with RP [7, 8]. Previously, a genetic study was performed to determine the distribution of causative genes; however, the number of subjects in the study was relatively small, and therefore, the ability of the findings to represent the general genetic distribution in Korean patients with RP is limited [9]. In the current study, we identified genetic profiles and associated clinical characteristics for several Korean patients with RP.

## 2. Materials and Methods

### 2.1. Patients

This retrospective study included patients who were clinically diagnosed with RP at a single tertiary clinic (Asan Medical Center in Seoul, Korea). This clinic is one of the largest centers in South Korea and has a nationwide referral area. Patients included in this study underwent molecular analyses using targeted next-generation sequencing (NGS) between November 2018 and December 2019, and only those who continued undergoing regular ophthalmic examinations for at least one year from the first visit were included in the final analysis. Genomic DNA was extracted from the peripheral blood of the patients and targeted NGS was performed using the Ion Torrent S5XL™ platform (Thermo Fisher, Waltham, MA, USA) with a panel of 88 genes that were previously shown to be associated with RP (Supplementary eTable 1). The mean coverage depth of the targeted NGS was approximately 500-fold, with 99.2% of the coverage >20-fold. Variant calling, annotation, and prioritization were performed as previously described [7]. Verification of the identified variants was waived when the read depth was >100 reads and the allele frequency was 40%–60% [8]. All the detected variants were classified according to the American College of Medical Genetics and Genomics guidelines [9]. This study was conducted according to the tenets of the Declaration of Helsinki, and all the study-related data acquisitions were approved by the Institutional Review Board (IRB) of the Asan Medical Center (IRB No. 2020-0859). The requirement for written informed consent was waived by the review board owing to the retrospective nature of the study. The results of each individual's molecular analyses and ophthalmic examinations were recorded on an anonymized case report form that was verified by the IRB. The data recorded on these report forms were then used in the final analyses.

### 2.2. Ocular Examinations

To confirm the clinical diagnosis of RP, each patient was subjected to a detailed ophthalmological examination. A comprehensive clinical history, which included a family history of disease, first ocular disease symptoms, and the presence of associated systemic symptoms, was collected for each patient. Additionally, the mode of inheritance was determined for each patient through a pedigree analysis based on the patient's clinical history. The ophthalmological examinations, which were performed according to the standards of the International Society for Clinical Electrophysiology of Vision, included best-corrected visual acuity (BCVA) measurements using Snellen charts (which were converted to logarithm of the minimum angle of resolution (LogMAR) units for the statistical analyses), manifest refraction, slit-lamp biomicroscopy, dilated fundoscopy, widefield fundus photography, fundus autofluorescence (FAF) imaging (Optos, Dunfermline, UK), spectral-domain optical coherence tomography (SD-OCT; Spectralis; Heidelberg Engineering, Heidelberg, Germany), and full-field electroretinograms (ERG) (Roland-Consult, Brandenburg Germany). In addition, a static Humphrey visual field test (HFA 750I, Carl Zeiss Meditec, Dublin, CA, USA) and kinetic Goldmann perimetry test (Haag-Streit AG, Köniz, Switzerland) were completed for each patient.

The macular retinal thickness was measured using SD-OCT with a custom 20° × 20° volume acquisition protocol to obtain high-speed scans. The built-in software (version 3.0) allowed for the automated segmentation of the retinal thickness at the fovea (central retinal thickness). The presence of an epiretinal membrane (ERM) or cystoid macular edema (CME) in consecutive scanned images was also reviewed. An ERM was defined as the area showing hyperreflectivity above the internal limiting membrane surface, with or without foveal distortion. CME was defined as a region with hyporeflective cystic spaces crossing the fovea on one or more consecutive scans; severe CME was defined as hyporeflective cystic spaces contiguously spread over more than three consecutive scans on OCT. To determine the degree of photoreceptor degeneration, a horizontal scan through the fovea was used to evaluate the horizontal width of the residual ellipsoid zone (EZ) line, which was manually measured using calipers built into the OCT software. To investigate the visual deterioration progression, BCVA measurements were collected during the follow-up periods. All the clinical data and the propriety of the diagnosis were collected/confirmed by two retina specialists (Y. N. Kim; Y. J. Kim).

### 2.3. Statistical Analysis

The categorical and continuous data are expressed as numbers (percentages) and the mean ± standard deviation, respectively. The progression patterns of VA deterioration and photoreceptor disruption were determined using nonlinear mixed models in which both fixed and random effects were entered nonlinearly. Linear and exponential growth models were tested, and the best fit model was selected using the model fit information, that is, the Akaike information criterion. Differences in the causative mutation genes were investigated using the group-time interaction effect. All the tests were two-tailed, and *P* values <0.05 were considered significant. The statistical analyses were performed using SAS version 9.4 (SAS Institute Inc., Cary, NC, USA) and SPSS version 21.0 (SPSS, Inc., Chicago. IL, USA).

## 3. Results

### 3.1. Clinical Characteristics of 150 Korean Patients with RP

Of the 150 total patients from 144 probands with RP included in the study, 74 were male (49.3%) and 76 were female (50.7%). Their baseline characteristics are described in Table 1 and Supplementary eTable 2. All the patients were of Korean ethnicity, and the median age at the ocular symptom onset was 20.0 years (range 4–67), whereas the median age at diagnosis was 43.0 years (range, 4–70). The most frequently reported first ocular symptom was night blindness (*N* = 118 (78.7%)), followed by decreased vision (*N* = 14 (9.3%)), constricted visual field (*N* = 11 (7.3%)), and dyschromatopsia (*N* = 4 (2.7%)).

The median age at which each patient was genetically tested was 49.0 years (range, 13–81). The median BCVA was 0.3 LogMAR in the right eye (range, 0.0–3.0) and 0.3 LogMAR in the left eye (range, 0.0–3.0) at the time of the genetic examination. All the patients showed a reduced response amplitude on the ERG. A generalized reduction of scotopic responses was noted among all the ERG responses, even for the patients with early-stage RP, and reduced cone, oscillatory, and flicker amplitudes (reflecting photoreceptor disruption) were observed for the patients who exhibited an advanced disease stage. The implicit response times were prolonged for most of the patients, except for a few with early-stage RP. During the fundus examinations, retinal degeneration, including pigmentary retinopathy, bone spicule pigments, and, rarely, clumps of pigment, and retinal vessel attenuation were found in all the patients. Hypoautofluorescence propagation corresponding to RPE atrophy was present around the vascular arcade and peripheral retina in the FAF images for all the patients. Additionally, the FAF images often revealed paracentral hyperautofluorescence rings or arcs (102/150 (68.0%)). It exhibits an abnormal accumulation of lipofuscin in the RPE due to apoptosis of external segmental dysplasia [10, 11], reflecting the transition line between abnormal and normal retinal architectures. These autofluorescence patterns were seen in the patients with VF loss. The patients for whom paracentral hyperautofluorescence rings or arcs appeared in the FAF images exhibited paracentral scotoma with a VF central island, which gradually contracted and eventually disappeared with the propagation of peripheral scotoma. ERM and CME were found in the OCT images for 54.7% and 30.0% of the patients with RP, respectively, and 46.7% (21/45) of the patients with CME showed severe CME.

### 3.2. Mutational Spectrum and Variant Analyses of 150 Korean Patients with RP

**Figure 1:**
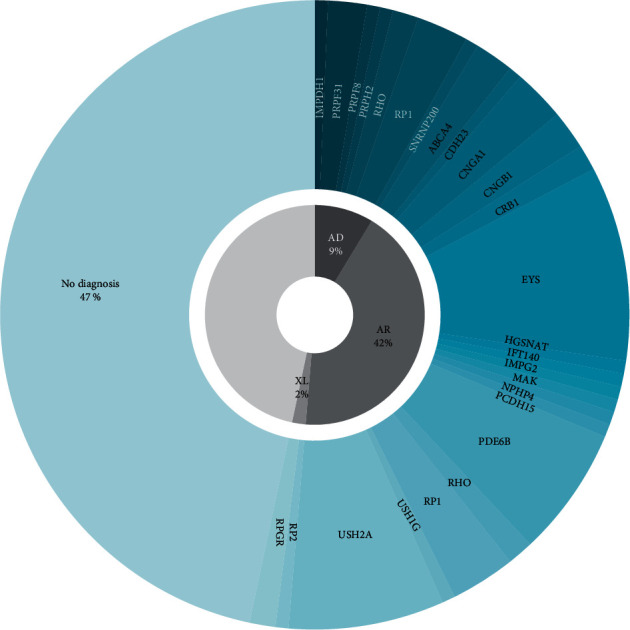
A mutational spectrum of the 150 Korean patients with retinitis pigmentosa. AD, autosomal dominant; AR, autosomal recessive; XL, X-linked inheritance.

Among the 144 probands, 77 (53.5%) possessed 82 variants in 24 genes that are responsible for causing RP. Among these 77 probands, 26 family members from 10 probands underwent segregation tests. Of the 82 detected variants, 28 (34.1%) were pathogenic, 23 (28.0%) were likely pathogenic, and 31 (37.8%) were variants of uncertain significance. The mutational spectrum of the RP-causative genes and their variants is shown in Figure 1, Table 2, and Supplementary eTable 3. Autosomal recessive variants accounted for the majority (*N* = 64 (44.4%)) of the cases, followed by autosomal dominant (*N* = 10 (6.9%)) and X-linked recessive variants (*N* = 3 (2.1%)). Four of the patients (5.2%) presented with syndromic RP. One patient had chronic kidney disease from nephron phthisis and carried an NPHP4 variant. Three patients had hearing loss and were diagnosed with Usher syndrome, and they independently carried CDH23, PCDH15, and USH1G variants. Of note, the four most frequently affected genes were EYS (*N* = 15 (10.4%), NM_001142800.1), USH2A (*N* = 12 (8.3%), NM_001142800.1), PDE6B (*N* = 9 (6.3%), NM_000283.3), and RP1 (*N* = 8 (5.6%), NM_006269.1), which accounted for 56.1% of the total pathogenic variants identified. In addition, 26 novel variants were identified, and they are listed in Table 2 and Supplementary eTable 3. Among these novel variants, two (7.7%; PRPF8-c.6902 C > T and USH1G-c.164 + 5G > A) were confirmed using segregation analysis.

### 3.3. Clinical Characteristics among the Four Major RP-Causative Genes

**Figure 2:**
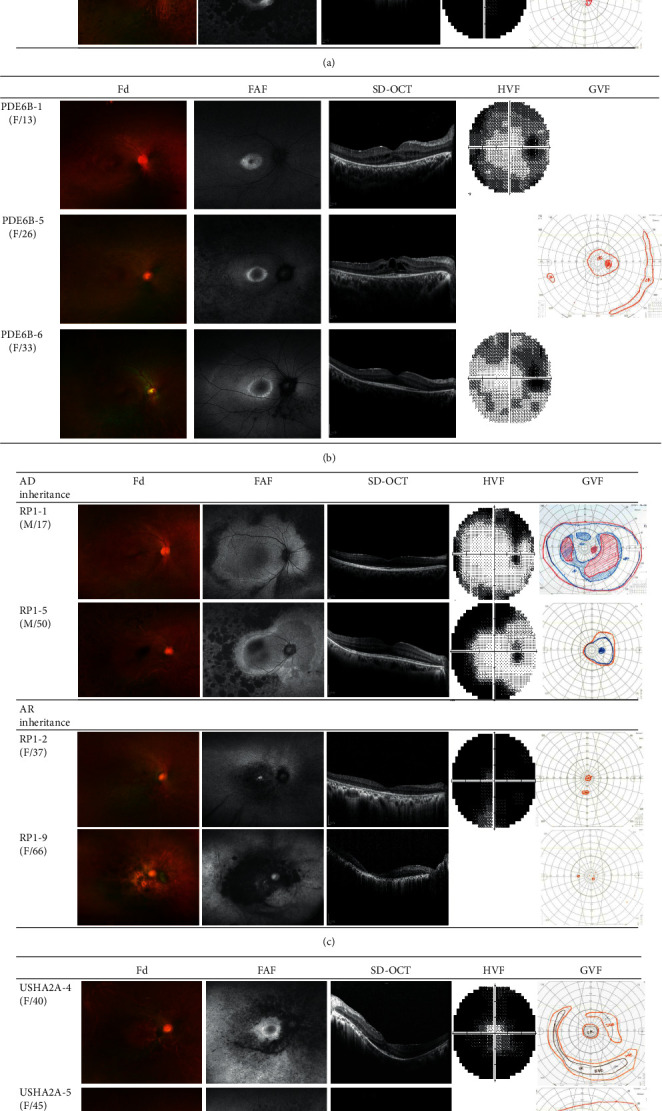
Disease progression patterns in retinitis pigmentosa patients carrying variants of four major causative genes. Disease progression patterns in (a) EYS-related RP: coarse pigmentation around the major vascular arcades and peripheral EZ disruption concomitant with peripheral VF constriction; (b) PDE6B-related RP: a bull's eye pattern of FAF consistent with paracentral scotoma. CME was frequently observed. (c) RP1-related RP: peripheral demarcated hyperautofluorescence lines and aggregation of pigments concomitant with a paracentral ring-shaped scotoma in the VF were characteristic features of AD RP1-RP. Perivascular pigmentation with macular atrophy was prominent in AR RP1-RP. (d) USH2A-related RP: fine pigmentation around the vascular arcade combined with paracentral scotoma in the VF. An ERM was found frequently. The images are presented in order of increasing patient age. RP, retinitis pigmentosa; EZ, ellipsoid zone; VF, visual field; CME, cystoid macular edema; ERM, epiretinal membrane; AD, autosomal dominant; AR, autosomal recessive; Fd, fundus photography; FAF, fundus autofluorescence; SD-OCT, spectral-domain optical coherence tomography, HVF, Humphrey visual field test, GVF; Goldmann visual field test.

Phenotypic differences among the patients carrying variants of any of the four major causative genes are described in Figure 2 and Table 1. For the patients with variants of EYS, coarse pigmentation that worsened with aging was prominent around the major vascular arcades (*N* = 12 (80.0%)), and peripheral EZ constriction concomitant with peripheral visual field constriction was also observed. For the patients with PDE6B variants, a bull's eye pattern of autofluorescence with central hypoautofluorescence surrounded by macular hyperautofluorescence was dominant (*N* = 7 (70.0%)). This finding was consistent with the pattern of paracentral scotoma and remaining central vision observed in the VF tests. Pigmentary degeneration occurred around the midperipheral retina and progressed toward the perivascular area. In addition, CME was more frequently observed in the patients with PDE6B variants (*N* = 5 (50.0%)) than in the other groups. Within the group of patients with variants of RP1, peripheral demarcated hyperautofluorescence lines and progressive pigment aggregation with age were the characteristic features of the patients with an autosomal dominant mode of inheritance (*N* = 4 (44.4%)). A paracentral ring-shaped scotoma was observed in the VF tests, which is in line with these funduscopic findings. However, perivascular pigmentation with macular atrophy was prominent in the patients carrying autosomal recessive RP1 variants (*N* = 5 (55.6%)). The absence or scarcity of typical RP-related hyperpigmentation at adolescence or early adulthood was noted in some of the patients with RP1 and USH2A variants. Finally, the patients with USH2A variants had relatively fine pigmentation around the vascular arcade combined with paracentral scotoma. They also had an ERM (*N* = 9 (75.0%)) and parafoveal EZ disruption more frequently than the patients in the other groups.

### 3.4. Disease Progression in Patients with RP Carrying Variants of Four Major Causative Genes

**Figure 3:**
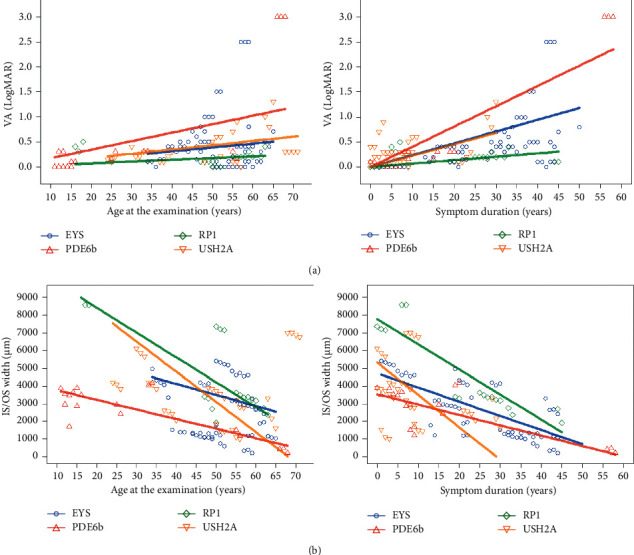
Changes in the visual acuity and photoreceptor layers in the patients with retinitis pigmentosa carrying variants of four causative genes during the follow-up periods. Changes in the (a) visual acuity and (b) width of the photoreceptor layers during the follow-up periods. The trend lines were drawn based on the patients' ages at the time of the examination and the symptom duration (defined as the period from the first symptoms to the age of the patient at the time of the examination). VA, visual acuity; LogMAR, logarithm of the minimum angle of resolution; IS/OS, photoreceptor inner segment/outer segment.

To compare the disease progression rates among the patients with RP carrying various gene mutations, changes in the BCVA and EZ bandwidths were analyzed during the follow-up periods for the patients carrying variants of the four major causative genes (Table 3 and Figure 3). Only the patients with the peripheral degeneration form of RP1-associated RP with pericentral degeneration were included in this analysis, as autosomal recessive RP1-associated RP primarily damages the macula [12]. During the follow-up duration (median 2.0 years, range: 1.0–20.0 years), the median BCVA reduction was 0.0 LogMAR (range: −0.2 to 1.0 LogMAR). The BCVA analysis trend based on age or symptom duration was analyzed using linear growth curve models (Figure 3(a)). Patients with PDE6B variants showed a BCVA reduction rate of 0.2 LogMAR/10 years during the follow-up periods. This rate was slightly faster than that of the other patients carrying other gene variants, which varied between 0.0 and 0.1 LogMAR/10 years, although the difference was not statistically significant. Regarding the onset of ocular symptoms, as recorded from the clinical histories, the patients with a PDE6B variant were also expected to experience the most rapid BCVA exacerbations, with a rate of 0.4 LogMAR/10 years from the time the first ocular symptom occurred. The functional deterioration rates, which were based on age and symptom duration, varied across the groups carrying variants of different causative genes; these differences were shown to be significant using the group-time interaction effect (Figure 3(a), *P*=0.014 and <0.001, resp.). Retinal structural changes were also assessed, and good interobserver reproducibility was obtained (intraclass correlation coefficient = 0.990, *p* < 0.001). The median intact EZ bandwidth shortening for the patients carrying variants of the four major genes was −421.5 *μ*m (range: −2295.0 to 158.0 *μ*m) during the follow-up period. The comparison of the EZ disruption rate was performed using linear growth curve models (Figure 3(b)). Patients with USH2A variants had the fastest shortening rate among all the patients carrying variants of the four major causative genes, that is, −170.4 *μ*m/year based on age and −182.8 *μ*m/year based on symptom duration.

## 4. Discussion

### 4.1. Mutational Spectrum and Variant Analyses of 150 Korean Patients with RP

In this study, we identified 82 variants from 24 causative genes in 77/144 probands using targeted NGS. Autosomal recessive inheritance accounted for the largest proportion of RP cases (44.4%), followed by autosomal dominant (6.9%) and X-linked inheritance (2.1%); this overall pattern was similar to that observed in previous studies of individuals of Western and Eastern ethnicities [13, 14]. Disease-causing variants of EYS (both pathogenic and likely pathogenic) were the most frequent cause of RP in this study (10.4%), followed by USH2A (8.3%), PDE6B (6.3%), and RP1 (5.6%). These results are in contrast with those from multiple studies of individuals of Western ethnicities, where USH2A accounted for most of the cases (up to 20–40% of the genetic composition) [13, 15, 16]. However, our results are consistent with those from other studies conducted in the East Asian region, where EYS variants are frequently found. EYS variants account for 20%–30% of Japanese RP cases, whereas <10% are caused by USH2A variants [14, 17]. These distributions are also similar to those reported in patients of Korean ethnicity [18]. However, in Chinese and Taiwanese populations, the genetic distributions are similar to those observed in individuals of Western or European ethnicities, where USH2A accounts for the majority of RP cases (20%–40%), and the proportion of EYS variants among patients with RP is <10% [19, 20]. These genetic distribution patterns can be explained by the migration history and genetic admixture of the East Asian population. A previous study demonstrated that the genetic differences among Han Chinese, Japanese, and Korean populations were smaller than those between Mongolian and European populations [21]. These genetic differences are consistent with the geographic distribution of the populations, which can also explain the results of the current study.

In this study, syndromic RP cases only accounted for 5.2% of all the detected cases; this proportion is lower than that previously reported in a population-based syndromic RP study (approximately 20–30%) [2]. Among the genes known to cause syndromic RP that were included in the gene panel used in our analysis, ALMS1, ARL6, BBS2, CLRN1, CNNM4, FLVCR1, HARS, MYO7A, NPHP4, OFD1, PANK2, PCDH15, TTC8, TTPA, USH1G, and USH2A and the genes causing Usher syndrome (CLRN1, MYO7A, PCDH15, and PDZD7; 1–9/100,000 globally [22–24]) and nephron phthisis (NPHP4; approximately 1/50,000 globally [25, 26]) are known to have a relatively higher prevalence among patients with syndromic RP. However, genes related to other syndromic disorders with a lower prevalence (<1/1,000,000 worldwide), including Alström syndrome (ALSM1; 1–9/1,000,000 [27]), Bardet-Biedl syndrome (ARL6, BBS2, TTC8; only a few reported cases in East Asia [28]), and Jalili syndrome (CNNM4; less than 1/1,000,000 [29]), were likely not present in this relatively small study population.

### 4.2. Clinical Characteristics of 150 Korean Patients with RP

In the present study, we investigated the mutational spectrum of Korean patients with RP and correlated the results with their phenotypic characteristics. The patient clinical histories revealed that the patients experienced their first ocular symptom at a median age of 18.0 years (range: 4.0–61.0) and were diagnosed with RP at a median age of 41.0 years (range, 10.0–68.0). Considering that the age of RP onset varies widely according to the different causative genes, the overall age distributions of the disease onset and diagnosis in this study were generally consistent with those reported previously [30]. Of the four major causative genes studied here, the patients with PDE6B variants experienced their first symptom at an earlier age (median age-12.0 years), whereas patients carrying EYS, RP1, and USH2A variants experienced their first ocular symptom in late adolescence or adulthood (median ages 20.0, 30.0, and 37.5 years, resp.). Compared to previous studies that grouped patients with RP into early-onset and late-onset groups, the distributions of the onset ages in our study showed similar trends [19, 31].

Interestingly, our results also showed that there was a pattern of morphological progression of pigment aggregation or EZ junctional defects in patients with specific causative gene variants. RP symptom severity varies because of the variable effects of gene variants and environmental factors [31], although the RP clinical triad (bone spicule pigmentation, attenuated retinal vessels, and optic nerve head pallor) has been noted in most reported cases [31, 32]. Characteristic fundus presentations correlated with concomitant peripheral visual field constriction for all the patients carrying one of the four major causative genes. In contrast, the typical pigmentary degeneration that occurs around the midperipheral retina and round clumps of pigmentation were only found in the patients with EYS or RP1 variants. Features of RP sine pigmento were also noted in the patients with RP1 and USH2A variants, which is consistent with previous reports [33, 34]. These heterogeneous funduscopic features indicate that an RP diagnosis cannot be ruled out simply because of the absence of typical pigmentation, especially in younger patients.

The presence of an ERM and CME also differs based on the causative gene involved. In our patient cohort, an ERM was most frequently observed in the patients carrying USH2A variants (75.0%). This is higher than the previously reported idiopathic ERM rate across different populations (1–28.9%) [35] or studies of patients with RP (5–60%) [36]. Considering that the patients with USH2A variants in this study were older, the ERM frequency could be explained by the age of the patients, as increased age is a risk factor for ERM development [35]. Meanwhile, CME was more commonly found in the patients with PDE6B variants (50.0%) than in the patients with other gene variants; this prevalence is relatively higher than that described in previous studies (26.9–50.9%) [36–38]. Although the etiology of CME in patients with PDE6B mutations is not fully understood, several hypotheses have been proposed, including Müller cell dysfunction [39].

To quantify the functional and anatomical progression of RP during the follow-up periods, we performed an analysis using a linear growth curve model. According to this analysis, the patients with PDE6B variants showed the fastest BCVA change rate, whereas the patients with RP1 variants showed the slowest rate. However, the EZ disruption rate was higher for the patients with USH2A and RP1 variants and lower for the patients with PDE6B and EYS variants. Despite the presence of CME in the patients with PDE6B variants, there was no direct correlation between CME and the residual EZ bandwidth or visual field, which is in line with previous studies showing that the existence of CME itself is not necessarily associated with decreased visual function [39–41]. We assumed that the central visual function was rapidly impaired due to disrupted macular photoreceptor integrity and subsequent foveal atrophy as the CME regressed in the patients with advanced RP, such as in those carrying PDE6B variants. However, for the patients with RP1 variants showing periphery dominant deterioration, BCVA disruption was relatively slow despite the fast overall EZ disruption. This slow central vision deterioration for the patients with RP1 variants can be explained by the preservation of their foveal photoreceptors as the demarcation line is formed around the major vascular arcades. Additionally, the slower EZ disruption rate acts as microperimetric biofeedback in the fovea throughout the patients' lifetime; consequently, this improves the fixation stability of both eyes, as reported previously [42].

### 4.3. Limitations of This Study

This single-center retrospective study has several limitations. First, the cohort was relatively small and was followed up over a short period of at least one year to evaluate the degenerative characteristics associated with each causative gene. Nevertheless, we attempted to minimize the effect of this limitation by using long-term observational data to develop nonlinear mixed models for assessing BCVA and EZ bandwidth changes to track disease progression. Although the number of patients was small, this did not result in any difficulty analyzing the nonlinear mixed model-given average of 4.0 repeated measurements per patient during the follow-up duration. Second, there was a lack of segregation analyses for the family data. Among the 80 patients with identified causative genes, only 26 members from 10 families were included in this study. To compensate for this limitation, we reviewed the population/frequency and in silico data to show the pathogenicity of the causative genes. Nevertheless, a comprehensive study including asymptomatic family members should be carried out to clarify the inheritance patterns and penetrance of the variants. Third, there may be causative genes that were not detected due to the limited number of genes included in the gene panel used in this study. We designed a preliminary panel of 88 RP-associated genes; this panel did not contain some genes that have been identified in previous studies to be associated with inherited retinal diseases [30, 43]. Although our test panel contained a relatively small number of causative genes, the results of our genetic analysis confirmed that the panel was not inferior to that used in other studies to detect frequently found RP-causative genes described in the mutational spectrums from the Discussion section in this report. Further analyses using additive gene panels or whole exome sequencing are planned to compensate for this limitation. Despite these shortcomings, we were able to categorize the clinical characteristics of major causative genes in Korean patients with RP. In addition, we quantified the rate of disease progression, BCVA, and OCT parameters, using longitudinal clinical data.

## 5. Conclusion

The results of this study have highlighted 24 RP-causative genes that were identified in 77 probands using targeted NGS, and the variants were correlated with the patient's clinical characteristics. By correlating the genetic analyses results with the clinical data from functional and structural assessments, we were able to describe RP disease progression that is specific to four major causative genes. The subgroup description of the four major RP-causative genes may provide valuable information that can aid in the early diagnosis and clinical prediction of RP. These results also highlight the need to conjugate targeted NGS data and clinical symptoms in clinical practice.

## Figures and Tables

**Table 1:** Baseline clinical characteristics of 150 Korean patients with retinitis pigmentosa who were grouped according to causative genes identified through targeted next-generation sequencing.

Gene	Inheritance	Patients/family no., *n*/*n*	Sex	Clinical history, years (range)			BCVA, LogMAR (range)		OCT parameters		
			M : F	Age at first symptom onset	Age at diagnosis	Age at genetic examination	OD	OS	ERM, *n* (%)	CME, *n* (%)	Width of EZ band, *μ*m (range)
EYS	AR	15/15	4 : 11	20.0 (7.0–51.0)	48.0 (34.0–62.0)	57.0 (37.0–66.0)	0.3 (0.0–3.0)	0.2 (0.0–3.0)	8 (53.3)	5 (33.0)	2660.0 (602.0–5180.0)

PDE6B	AR	10/9	5 : 5	12.0 (4.0–47.0)	18.0 (11.0–55.0)	29.5 (13.0–67.0)	0.2 (0.0–3.0)	0.2 (0.0–0.5)	3 (30.0)	5 (50.0)	2644.0 (328.0–4734.0)

RP1	AD/AR	9/8	4 : 5	30.0 (5.0–50.0)	48.0 (17.0–56.0)	50.0 (17.0–66.0)	0.2 (0.0–3.0)	0.2 (0.0–3.0)	6 (66.7)	1 (11.1)	2380.0 (371.0–8583.0)

USH2A	AR	12/12	6 : 6	37.5 (15.0–61.0)	40.5 (24.0–68.0)	50.5 (26.0–71.0)	0.3 (0.0–0.9)	0.3 (0.0–0.7)	9 (75.0)	4 (33.3)	2380.5 (1010.0–6750.0)

Others		34/33	19 : 15	13.0 (5.0–55.0)	38.0 (13.0–62.0)	44.5 (17.0–64.0)	0.3 (0.0–3.0)	0.4 (0.0–3.0)	22 (64.7)	5 (14.7)	966.5 (259.0–5849.0)

Variants detected		80/77	38 : 42	18.0 (4.0–61.0)	41.0 (10.0–68.0)	49.0 (13.0–72.0)	0.3 (0.0–3.0)	0.3 (0.0–3.0)	48 (61.0)	20 (25.6)	2057.0 (259.0–8583.0)

Not identified		70/67	36 : 34	24.0 (4.0–67.0)	45.5 (4.0–70.0)	49.0 (14.0–81.0)	0.3 (0.0–3.0)	0.3 (0.0–3.0)	34 (48.6)	25 (35.7)	

Total		150/144	74 : 76	20.0 (4.0–67.0)	43.0 (4.0–70.0)	49.0 (13.0–81.0)	0.3 (0.0–3.0)	0.3 (0.0–3.0)	82 (54.7)	45 (30.0)	

BCVA, best-corrected visual acuity; LogMAR, logarithm of the minimum angle of resolution; OCT, optical coherence tomography; M, male; F, female; OD, oculus dexter; OS, oculus sinister; ERM, epiretinal membrane; CME, cystoid macular edema; EZ, ellipsoid zone; AR, autosomal recessive; AD, autosomal dominant.

**Table 2:** Descriptions of the four major causative genes and their variants in Korean patients with retinitis pigmentosa.

Subject no.	Causative gene	NM number	Chromosome	HGVSDNA change	HGVS protein change	Zygosity	Inheritance	Mutation type	ACMG criteria	
EYS-1	EYS	NM_001142800.1	6	c.4957dup	p.Ser1653fs	Hetero	AR	Nonsense	P	PVS1PM2PP5
EYS-1	EYS	NM_001142800.1	6	c.2528G>A	p.Gly843Glu	Hetero	AR	Missense	LP	PM1PM2PP3PP5
EYS-2	EYS	NM_001142800.1	6	c.4957dup	p.Ser1653fs	Homo	AR	Nonsense	P	PVS1PM2PP5
EYS-3	EYS	NM_001142800.1	6	c.4957dup	p.Ser1653fs	Hetero	AR	Nonsense	P	PVS1PM2PP5
EYS-3	EYS	NM_001142800.1	6	c.7394C>G	p.Thr2465Ser	Hetero	AR	Missense	VUS^*b*^	PM1PM2PP3
EYS-4	EYS	NM_001142800.1	6	c.4957dup	p.Ser1653fs	Hetero	AR	Nonsense	P	PVS1PM2PP5
EYS-4	EYS	NM_001142800.1	6	c.6557G>A	p.Gly2186Glu	Hetero	AR	Missense	LP	PM1PM2PP3PP5
EYS-5	EYS	NM_001142800.1	6	c.8805C>A	p.Tyr2935Ter	Hetero	AR	Nonsense	P	PVS1PM2PM5
EYS-5	EYS	NM_001142800.1	6	c.6557G>A	p.Gly2186Glu	Hetero	AR	Missense	LP	PM1PM2PP3PP5
EYS-6	EYS	NM_001142800.1	6	c.6557G>A	p.Gly2186Glu	Hetero	AR	Missense	LP	PM1PM2PP3PP5
EYS-6	EYS	NM_001142800.1	6	c.525_527del	p.Glu176del	Hetero	AR	In-frame deletion	VUS^*b*^	PM3
EYS-7	EYS	NM_001142800.1	6	c.2641 + 1G>A		Hetero	AR		P^*a*^	PVS1PM2PP3
EYS-7	EYS	NM_001142800.1	6	c.586A>C	p.Lys196Gln	Hetero	AR	Missense	VUS^*b*^	PM1PM2PP5
EYS-8	EYS	NM_001142800.1	6	c.4957dup	p.Ser1653fs	Homo	AR	Nonsense	P	PVS1PM2PP5
EYS-9	EYS	NM_001142800.1	6	c.8805C>A	p.Tyr2935Ter	Hetero	AR	Nonsense	P	PVS1PM2PP5
EYS-9	EYS	NM_001142800.1	6	c.525_527del	p.Glu176del	Hetero	AR	In-frame deletion	VUS^*b*^	PM2PM4
EYS-10	EYS	NM_001142800.1	6	c.8805C>A	p.Tyr2935Ter	Hetero	AR	Nonsense	P	PVS1PM2PP5
EYS-10	EYS	NM_001142800.1	6	c.1963G>T	p.Gly655Ter	Hetero	AR	Nonsense	P^*a*^	PVS1PPMPP3
EYS-11	EYS	NM_001142800.1	6	c.4957dup	p.Ser1653fs	Hetero	AR	Nonsense	P	PVS1PM2PP5
EYS-11	EYS	NM_001142800.1	6	c.8805C>A	p.Tyr2935Ter	Hetero	AR	Nonsense	P	PVS1PM2PP5
EYS-12	EYS	NM_001142800.1	6	c.1963G>T	p.Gly655Ter	Hetero	AR	Nonsense	P	PVS1PM2PP3
EYS-12	EYS	NM_001142800.1	6	c.9368delA	p.Asn3123fs	Hetero	AR	Nonsense	LP	PVS1PM2
EYS-13	EYS	NM_001142800.1	6	c.2528G>A	p.Gly843Glu	Hetero	AR	Missense	LP	PM1PM2PP3PP5
EYS-13	EYS	NM_001142800.1	6	c.6571 + 6T>A		Hetero	AR	Missense	VUS^*a*,*b*^	PM2PP3
EYS-14	EYS	NM_001142800.1	6	c.2528G>A	p.Gly843Glu	Hetero	AR	Missense	LP	PM1PM2PP3PP5
EYS-14	EYS	NM_001142800.1	6	c.7492G>C	p.Ala2498Pro	Hetero	AR	Missense	VUS^*a*,*b*^	PM1PM2PP3
EYS-14	EYS	NM_001142800.1	6	c.1382G>A	p.Cys461Tyr	Hetero	AR	Missense	VUS^*b*^	PM2
EYS-15	EYS	NM_001142800.1	6	c.4957dup	p.Ser1653fs	Hetero	AR	Nonsense	P	PVS1PM2PP5
EYS-15	EYS	NM_001142800.1	6	c.525_527del	p.Glu176del	Hetero	AR	In-frame deletion	VUS(LP)	PM2PM4PM3

PDE6B-1	PDE6B	NM_000283.3	6	c.1669C>T	p.His557Tyr	Homo	AR	Missense	LP	PM1PM2PP3PP5
PDE6B-2	PDE6B	NM_000283.3	6	c.1488del	p.Thr497fs	Hetero	AR	Nonsense	P	PVS1PM2PP5
PDE6B-2	PDE6B	NM_000283.3	6	c.1669C>T	p.His557Tyr	Hetero	AR	Missense	LP	PM1PM2PP3PP5
PDE6B−3^∗^	PDE6B	NM_000283.3	6	c.2395C>T	p.Arg799Ter	Hetero	AR	Nonsense	P	PVS1PM2PP3PP5
PDE6B−3	PDE6B	NM_000283.3	6	c.1712C>T	p.Thr571Met	Hetero	AR	Missense	VUS	PM1PM2PP5
PDE6B-4^∗^	PDE6B	NM_000283.3	6	c.2395C>T	p.Arg799Ter	Hetero	AR	Nonsense	P	PVS1PM2PP3PP5
PDE6B-4	PDE6B	NM_000283.3	6	c.1712C>T	p.Thr571Met	Hetero	AR	Missense	VUS	PM1PM2PP5
PDE6B-5	PDE6B	NM_000283.3	6	c.1547T>C	p.Leu516Pro	Hetero	AR	Missense	LP	PM1PM2PP3PP5
PDE6B-5	PDE6B	NM_000283.3	6	c.1669C>T	p.His557Tyr	Hetero	AR	Missense	LP	PM1PM2PP3PP5
PDE6B-6	PDE6B	NM_000283.3	6	c.1669C>T	p.His557Tyr	Homo	AR	Missense	LP	PM1PM2PP3PP5
PDE6B-7	PDE6B	NM_000283.3	6	c.712del	p.Val238fs	Hetero	AR	Frameshift	LP^a^	PVS1PM2
PDE6B-7	PDE6B	NM_000283.3	6	c.2492C>T	p.Ala831Val	Hetero	AR	Missense	VUS^,*b*^	PM1PM2BP4
PDE6B-8	PDE6B	NM_000283.3	6	c.1669C>T	p.His557Tyr	Homo	AR	Missense	LP	PM1PM2PP3PP5
PDE6B-9	PDE6B	NM_000283.3	6	c.1604T>A	p.Ile535Asn	Homo	AR	Missense	LP	PM1PM2PP3PP5
PDE6B-10	PDE6B	NM_000283.3	6	c.1669C>T	p.His557Tyr	Homo	AR	Missense	LP	PM1PM2PP3PP5

RP1-1	RP1	NM_006269.1	8	c.256C>A	p.Pro86Thr	Hetero	AD/AR	Missense	VUS^*b*^	PM1PM2PP3
RP1-2	RP1	NM_006269.1	8	c.5797C>T	p.Arg1933Ter	Homo	AD/AR	Nonsense	P	PVS1PM2PP3PP5
RP1-3^∗^	RP1	NM_006269.1	8	c.6181del	p.Ile2061fs	Hetero	AD/AR	Coding sequence variant	VUS	PM2PP5
RP1-4^∗^	RP1	NM_006269.1	8	c.6181del	p.Ile2061fs	Hetero	AD/AR	Coding sequence variant	VUS	PM2PP5
RP1-5	RP1	NM_006269.1	8	c.4196del	p.Cys1399fs	Hetero	AD/AR	Frameshift	P	PVS1PM2PP5
RP1-5	RP1	NM_006269.1	8	c.6353G>A	p.Ser2118Asn	Hetero		Missense	VUS^*b*^	PM2PP5
RP1-6	RP1	NM_006269.1	8	c.5797C>T	p.Arg1933Ter	Hetero	AD/AR	Nonsense	P	PVS1PM2PP3PP5
RP1-7^∗^	RP1	NM_006269.1	8	c.2296C>T	p.Gln766Ter	Hetero	AD/AR	Nonsense	P	PVS1PM2PP3
RP1-7	RP1	NM_006269.1	8	c.5913C>A	p.Asn1971Lys	Hetero		Missense	VUS	PM2BP1
RP1-8	RP1	NM_006269.1	8	c.2238_2239del	p.Ser747Ter	Hetero	AD/AR	Nonsense	LP	PVS1PM2
RP1-9	RP1	NM_006269.1	8	c.4196del	p.Cys1399fs	Hetero	AD/AR	Frameshift	P	PVS1PM2PP5
RP1-9	RP1	NM_006269.1	8	c.5797C>T	p.Arg1933Ter	Hetero		Nonsense	P	PVS1PM2PP3PP5
RP1-9	RP1	NM_006269.1	8	c.6353G>A	p.Ser2118Asn	Hetero		Missense	VUS^*b*^	PM2PP5

USHA2A-1	USH2A	NM_001142800.1	1	c.8254G>A	p.Gly2752Arg	Hetero	AR	Missense	LP	PM1PM2PP3
USHA2A-1	USH2A	NM_001142800.1	1	c.451G>C	p.Ala151Pro	Hetero	AR	Missense	VUS^*a*,*b*^	PM1PM2BP4
USHA2A-2	USH2A	NM_001142800.1	1	c.6326-1G>T		Hetero	AR		P^*a*^	PVS1PM2PP3
USHA2A-2	USH2A	NM_001142800.1	1	c.11156G>A	p.Arg3719His	Hetero	AR	Missense	VUS^*b*^	PM1PM2PP5
USHA2A-3	USH2A	NM_001142800.1	1	c.2802T>G	p.Cys934Trp	Hetero	AR	Missense	LP	PM1PM2PP3PP5
USHA2A-3	USH2A	NM_001142800.1	1	c.11136_11137del	p.Gln3714fs	Hetero	AR	Frameshift	LP^*a*^	PVS1PM2
USHA2A-3	USH2A	NM_001142800.1	1	c.15518T>C	p.Leu5173Pro	Hetero	AR	Missense	VUS^*a*,*b*^	PM2PP3
USHA2A-4	USH2A	NM_001142800.1	1	c.8559-2A>G		Hetero	AR	Splice acceptor	P	PVS1PM2PP3PP5
USHA2A-4	USH2A	NM_001142800.1	1	c.2802T>G	p.Cys934Trp	Hetero	AR	Missense	LP	PM1PM2PP3PP5
USHA2A-5^∗^	USH2A	NM_001142800.1	1	c.2802T>G	p.Cys934Trp	Hetero	AR	Missense	LP	PM1PM2PP3PP5
USHA2A-5	USH2A	NM_001142800.1	1	c.13339A>G	p.Met4447Val	Hetero	AR	Missense	VUS^*b*^	PM1PM2
USHA2A-6	USH2A	NM_001142800.1	1	c.14287G>A	p.Gly4763Arg	Hetero	AR	Missense	LP	PM1PM2PP3PP5
USHA2A-6	USH2A	NM_001142800.1	1	c.1190T>A	p.Ile397Lys	Hetero	AR	Missense	VUS^*a*,*b*^	PM1PM2PP3
USHA2A-7	USH2A	NM_001142800.1	1	c.7046G>A	p.Trp2349Ter	Hetero	AR	Nonsense	P^*a*^	PVS1PM2PP3
USHA2A-7	USH2A	NM_001142800.1	1	c.2802T>G	p.Cys934Trp	Hetero	AR	Missense	LP	PM1PM2PP3PP5
USHA2A-8	USH2A	NM_001142800.1	1	c.2802T>G	p.Cys934Trp	Hetero	AR	Missense	LP	PM1PM2PP3PP5
USHA2A-8	USH2A	NM_001142800.1	1	c.8254G>A	p.Gly2752Arg	Hetero	AR	Missense	LP	PM1PM2PP3PP5
USHA2A-9	USH2A	NM_001142800.1	1	c.2802T>G	p.Cys934Trp	Homo	AR	Missense	LP	PM1PM2PP3PP5
USHA2A-10	USH2A	NM_001142800.1	1	c.1450C>T	p.Gln484Ter	Hetero	AR	Nonsense	P	PVS1PM2PP3
USHA2A-10	USH2A	NM_001142800.1	1	c.2802T>G	p.Cys934Trp	Hetero	AR	Missense	LP	PM1PM2PP3PP5
USHA2A-11	USH2A	NM_001142800.1	1	c.9258 + 1G>T		Hetero	AR	Splice donor	P	PVS1PM2PP3PP5
USHA2A-11	USH2A	NM_001142800.1	1	c.2802T>G	p.Cys934Trp	Hetero	AR	Missense	LP	PM1PM2PP3PP5
USHA2A-11	USH2A	NM_001142800.1	1	c.14557A>G	p.Met4853Val	Hetero	AR	Missense	VUS^*b*^	PM1PM2BP4
USHA2A-12	USH2A	NM_001142800.1	1	c.8559-2A>G		Hetero	AR	Splice acceptor	P	PVS1PM2PP3PP5
USHA2A-12	USH2A	NM_001142800.1	1	c.2802T>G	p.Cys934Trp	Hetero	AR	Missense	LP	PM1PM2PP3PP5
USHA2A-12	USH2A	NM_001142800.1	1	c.15178T>C	p.Ser5060Pro	Hetero	AR	Missense	VUS^*b*^	PM2

ACMG, American College of Medical Genetics and Genomics; HGVS, Human Genome Variation Society; P, pathogenic variant; LP, likely pathogenic variant; VUS, variant of unknown significance; AR, autosomal recessive; AD, autosomal dominant; XL, X-linked; hetero, heterozygote; homo, homozygote. ^∗^Patients who underwent segregation testing; ^*a*^novel variants; ^*b*^VUS confirmed by definitive retinitis pigmentosa phenotype.

**Table 3:** Disease progression in patients with retinitis pigmentosa carrying variants of the four major causative genes.

Gene	Inheritance	No., *n*	Follow-up duration, years (range)	Change in BCVA					Shortening of EZ band, *μ*m (range)	Change in EZ band			
				Deterioration of BCVA, logMAR (range)	Age at exam, years^*a*^		Symptom duration, years^*b*^			Age at exam, years^*a*^		Symptom duration, years^*b*^	
					Beta	*P* value^*c*^	Beta	*P* value^c^		Beta	*P* value^c^	Beta	*P* value^c^
EYS	AR	15	2.0 (1.0–20.0)	0.1 (−0.1−1.0)	0.01	0.0003	0.02	<0.0001	−555.0 (−1615.0−158.0)	−62.28	0.0002	−80.09	<0.0001
PDE6B	AR	10	1.5 (1.0–4.0)	0.0 (−0.2−0.2)	0.02	0.0342	0.04	<0.0001	−247.0 (−1239.0−135.0)	−54.26	0.0105	−58.60	0.0299
RP1	AD/AR∗	4/5∗	2.0 (2.0–8.0)	0.1 (0.0–0.2)	0.00	0.0851	0.01	0.0590	−35.0 (−1243.0—1.0)	−140.22	<0.0001	−141.80	<0.0001
USH2A	AR	12	2.0 (1.0–9.0)	0.0 (−0.1 − 0.5)	0.01	0.0002	0.02	0.0039	−676.2 (−1676.0 − 0.0)	−170.37	<0.0001	−182.76	<0.0001
Four major causative genes		41/42∗	2.0 (1.0–20.0)	0.0 (−0.2 − 1.0)					−421.5 (−2295.0 − 158.0)				
Group-time interaction effect^*d*^						0.0140		0.0006			0.0336		0.0050

BCVA, best-corrected visual acuity; EZ, ellipsoid zone; SD, standard deviation; LogMAR, logarithm of the minimum angle of resolution; AR, autosomal recessive; AD, autosomal dominant. ^*a*^Linear growth curve model: *y* = beta·*t* (model without intercept). ^*b*^Linear growth curve model: *y* = *a* + beta·*t*. ^*c*^*P* < 0.05 was considered significant. ^*d*^*P* value for interaction between groups, *P* < 0.05 was considered significant. ∗Only patients (five out of nine) with peripheral type RP1-associated RP were analyzed.

## Data Availability

The data used to support the findings of this study are included within the article and supplementary information files.

## Supplementary Materials

Supplementary Materials Supplementary eTable 1. Targeted next-generation sequencing panel consisted of 88 genes associated with retinitis pigmentosa. Supplementary eTable 2. Baseline clinical characteristics of 80 Korean patients with retinitis pigmentosa carrying variants in the detected causative genes. Supplement eTable 3. Causative genes and variants in 80 Korean patients with retinitis pigmentosa.

## References

[1] Hartong D. T., Berson E. L., Dryja T. P. (2006). Retinitis pigmentosa. *The Lancet*.

[2] Ferrari S., Di Iorio E, Barbaro V, Ponzin D, Sorrentino F. S, Parmeggiani F (2011). Retinitis pigmentosa: genes and disease mechanisms. *Current Genomics*.

[3] Sorrentino F. S., Gallenga C. E., Bonifazzi C., Perri P. (2016). A challenge to the striking genotypic heterogeneity of retinitis pigmentosa: a better understanding of the pathophysiology using the newest genetic strategies. *Eye*.

[4] Daiger S. P., Bowne S. J., Sullivan L. S. (2007). Perspective on genes and mutations causing retinitis pigmentosa. *Archives of Ophthalmology*.

[5] Daiger S. P., Sullivan P. L. S., RetNet P. S. J. B.. (2020). *Retinal Information Network*.

[6] Glöckle N., Kohl S., Mohr J. (2014). Panel-based next generation sequencing as a reliable and efficient technique to detect mutations in unselected patients with retinal dystrophies. *European Journal of Human Genetics*.

[7] Seo G. H., Park J. -Y., Kim S. (2019). *High Diagnostic Yield and Clinical Utility of WES for Patients with Undiagnosed Genetic Disorder by Automating Variant Interpretation*.

[8] Abecasis G. R., Altshuler D., Auton A. (2010). A map of human genome variation from population-scale sequencing. *Nature*.

[9] Richards S., Aziz N., Bale S. (2015). Standards and guidelines for the interpretation of sequence variants: a joint consensus recommendation of the American college of medical genetics and genomics and the association for molecular pathology. *Genetics in Medicine*.

[10] Lima L. H., Cella W., Greenstein V. C. (2009). Structural assessment of hyperautofluorescent ring in patients with retinitis pigmentosa. *Retina*.

[11] Robson A. G., Saihan Z., Jenkins S. A. (2006). Functional characterization and serial imaging of abnormal fundus autofluorescence in patients with retinitis pigmentosa and normal visual acuity. *British Journal of Ophthalmology*.

[12] Verbakel S. K., van Huet R. A. C., Den Hollander A. I. (2019). Macular dystrophy and cone-rod dystrophy caused by mutations in the RP1 Gene: extending the RP1 Disease spectrum. *Investigative Opthalmology & Visual Science*.

[13] Martin-Merida I., Avila-Fernandez A., Del Pozo-Valero M. (2019). Genomic landscape of sporadic retinitis pigmentosa. *Ophthalmology*.

[14] Oishi M., Oishi A., Gotoh N. (2014). Comprehensive molecular diagnosis of a large cohort of Japanese retinitis pigmentosa and Usher syndrome patients by next-generation sequencing. *Investigative Opthalmology & Visual Science*.

[15] Dias M. F., Joo K., Kemp J. A. (2018). Molecular genetics and emerging therapies for retinitis pigmentosa: Basic research and clinical perspectives. *Progress in Retinal and Eye Research*.

[16] Karali M., Testa F, Brunetti-Pierri R (2019). Clinical and genetic analysis of a European cohort with pericentral retinitis pigmentosa. *International Journal of Molecular Sciences*.

[17] Arai Y., Maeda A, Hirami Y (2015). Retinitis pigmentosa with EYS mutations is the most prevalent inherited retinal dystrophy in Japanese populations. *Journal of ophthalmology*.

[18] Kim M. S., Joo K, Seong M. W (2019). Genetic mutation profiles in Korean patients with inherited retinal diseases. *Journal of Korean Medical Science*.

[19] Gao F.-J., Li J.-K., Chen H. (2019). Genetic and clinical findings in a large cohort of Chinese patients with suspected retinitis pigmentosa. *Ophthalmology*.

[20] Chen Z. J., Lin K. H., Lee S. H. (2020). Mutation spectrum and genotype‐phenotype correlation of inherited retinal dystrophy in Taiwan. *Clinical & Experimental Ophthalmology*.

[21] Wang Y., Lu D., Chung Y.-J., Xu S. (2018). Genetic structure, divergence and admixture of Han Chinese, Japanese and Korean populations. *Hereditas*.

[22] Hope C. I., Bundey S., Proops D., Fielder A. R. (1997). Usher syndrome in the city of Birmingham---prevalence and clinical classification. *British Journal of Ophthalmology*.

[23] Rosenberg T., Haim M., Hauch A. M., Parving A. (1997). The prevalence of Usher syndrome and other retinal dystrophy-hearing impairment associations. *Clinical Genetics*.

[24] Espinós C., Millán J. M., Beneyto M., Nájera C. (1998). Epidemiology of usher syndrome in Valencia and Spain. *Public Health Genomics*.

[25] Potter D. E., Holliday M. A., Piel C. F., Feduska N. J., Belzer F. O., Oscar Salvatierra J. (1980). Treatment of end-stage renal disease in children: a 15-year experience. *Kidney International*.

[26] Ala-Mello S., Koskimies O., Rapola J., Kääriäinen H. (1999). Nephron phthisis in Finland: epidemiology and comparison of genetically classified subgroups. *European Journal of Human Genetics*.

[27] D. Marshall J., Maffei P., B. Collin G., K. Naggert J. (2011). Alstrom syndrome: genetics and clinical overview. *Current Genomics*.

[28] Hirano M., Satake W., Ihara K. (2015). The first nationwide survey and genetic analyses of Bardet-Biedl syndrome in Japan. *PLoS One*.

[29] Daneshmandpour Y., Darvish H., Pashazadeh F., Emamalizadeh B. (2019). Features, genetics and their correlation in Jalili syndrome: a systematic review. *Journal of Medical Genetics*.

[30] Pierrache L. H. M., Hartel B. P., Van Wijk E. (2016). Visual prognosis in USH2A-associated retinitis pigmentosa is worse for patients with usher syndrome type IIa than for those with nonsyndromic retinitis pigmentosa. *Ophthalmology*.

[31] Verbakel S. K., van Huet R. A. C., Boon C. J. F. (2018). Non-syndromic retinitis pigmentosa. *Progress in Retinal and Eye Research*.

[32] Schachat A. P., Wilkinson C. P., Hinton D. R., Sadda S. R., Wiedemann P. (2017). Ryan’s retina e-book. *Elsevier Health Sciences*.

[33] Chen X., Sheng X., Liu X. (2014). Targeted next-generation sequencing reveals novel USH2A mutations associated with diverse disease phenotypes: implications for clinical and molecular diagnosis. *PLoS One*.

[34] Ma L., Sheng X. L, Li H. P (2013). Identification of a novel p.R1443W mutation in RP1 gene associated with retinitis pigmentosa sine pigmento. *International Journal of Ophthalmology*.

[35] Bu S.-C., Kuijer R., Li X.-R., Hooymans J. M. M., Los L. I. (2014). Idiopathic epiretinal membrane. *Retina*.

[36] Liew G., Strong S., Bradley P. (2019). Prevalence of cystoid macular oedema, epiretinal membrane and cataract in retinitis pigmentosa. *British Journal of Ophthalmology*.

[37] Adackapara C. A., Sunness J. S., Dibernardo C. W., Melia B. M., Dagnelie G. (2008). Prevalence of cystoid macular edema and stability in oct retinal thickness in eyes with retinitis pigmentosa during a 48-week lutein trial. *Retina*.

[38] Makiyama Y., Oishi A., Otani A. (2014). Prevalence and spatial distribution of cystoid spaces in retinitis pigmentosa. *Retina*.

[39] Yeo J. H., Kim Y. J., Yoon Y. H. (2020). Optical coherence tomography angiography in patients with retinitis pigmentosa-associated cystoid macular edema. *Retina*.

[40] Kim Y. J., Joe S. G., Lee D.-H., Lee J. Y., Kim J.-G., Yoon Y. H. (2013). Correlations between spectral-domain OCT measurements and visual acuity in cystoid macular edema associated with retinitis pigmentosa. *Investigative Opthalmology & Visual Science*.

[41] Oishi A., Otani A., Sasahara M. (2009). Photoreceptor integrity and visual acuity in cystoid macular oedema associated with retinitis pigmentosa. *Eye*.

[42] Grenga P. L., Trabucco P., Meduri A., Fragiotta S., Vingolo E. M. (2013). Microperimetric biofeedback in a patient with oculocutaneous albinism. *Canadian Journal of Ophthalmology*.

[43] Huang X.-F., Huang F., Wu K.-C. (2015). Genotype-phenotype correlation and mutation spectrum in a large cohort of patients with inherited retinal dystrophy revealed by next-generation sequencing. *Genetics in Medicine*.

