# Supplementary material for: Genetic Profile and Associated Characteristics of 150 Korean Patients with Retinitis Pigmentosa
Source: J Ophthalmol. 2021 Oct 21;2021:5067271. doi: 10.1155/2021/5067271 (PMC8553513; doi:10.1155/2021/5067271)
Supplement: Supplementary Materials — Supplementary eTable 1. Targeted next-generation sequencing panel consisted of 88 genes associated with retinitis pigmentosa. Supplementary eTable 2. Baseline clinical characteristics of 80 Korean patients with retinitis pigmentosa carrying variants in the detected causative genes. Supplement eTable 3. Causative genes and variants in 80 Korean patients with retinitis pigmentosa. [file 5067271.f1.docx]

**Supplementary eTable 1.** Targeted next-generation sequencing panel consisted of 88 genes associated with retinitis pigmentosa.

| *ABCA4* | *AIPL1* | *ALMS1* | *ARL6* | *ATF6* | *BBS2* | *BEST1* | *C2orf71* | *CA4* | *CACNA1F* |
| --- | --- | --- | --- | --- | --- | --- | --- | --- | --- |
| *CACNA2D4* | *CDH23* | *CDHR1* | *CERKL* | *CLRN1* | *CNGA1* | *CNGB1* | *CNNM4* | *CRB1* | *CRX* |
| *DFNB31* | *DHDDS* | *EYS* | *FAM161A* | *FLVCR1* | *FSCN2* | *GUCA1A* | *GUCA1B* | *GUCY2D* | *HARS* |
| *HGSNAT* | *IDH3B* | *IFT140* | *IFT172* | *IL1A* | *IL1B* | *IMPDH1* | *IMPG2* | *KCNV2* | *KLHL7* |
| *LRAT* | *MAK* | *MFRP* | *MYO7A* | *NPHP4* | *NR2E3* | *NRL* | *OFD1* | *PANK2* | *PCDH15* |
| *PDE6A* | *PDE6B* | *PDE6C* | *PDE6G* | *PDZD7* | *PITPNM3* | *PROM1* | *PRPF3* | *PRPF31* | *PRPF6,* |
| *PRPF8* | *PRPH2* | *RBP3* | *RDH12* | *RGR* | *RHO* | *RIMS1* | *RLBP1* | *ROM1* | *RP1* |
| *RP2* | *RPE65* | *RPGR* | *RPGRIP1* | *RRM2B,* | *SAG* | *SEMA4A* | *SNRNP200* | *SPATA7* | *TOPORS* |
| *TTC8* | *TTPA* | *TULP1* | *UNC119* | *USH1G* | *USH2A* | *ZNF408* | *ZNF513* |  |  |

**Supplementary eTable 2.** Baseline clinical characteristics of 80 Korean patients with retinitis pigmentosa carrying variants in the detected causative genes.

| Gene | Inheritance | Patients/family No., n/n | Sex | Clinical history, years (range) | | | BCVA, LogMAR (range) | | OCT parameters | | |
| --- | --- | --- | --- | --- | --- | --- | --- | --- | --- | --- | --- |
|  |  |  | M : F | Age at first symptom onset | Age at diagnosis | Age at genetic examination | OD | OS | ERM, n (%) | CME, n (%) | Width of EZ band, µm (range) |
| *ABCA4* | AD/AR | 3/3 | 0 : 3 | 18.0 (14.0-20.0) | 43.0 (33.0-52.0) | 49.0 (43.0-62.0) | 1.5 (0.0-1.5) | 1.5 (0.0-2.5) | 3 (100.0) | 0 (0.0) | 506.0 (401.0-4120.0) |
| *CDH23* | AR/DR | 1/1 | 1 : 0 | 16.0 | 62.0 | 62.0 | 0.9 | 3.0 | 1 (100.0) | 0 (0.0) | 1030.0 |
| *CNGA1* | AR | 4/4 | 2 : 2 | 10.0 (5.0-14.0) | 46.0 (41.0-55.0) | 47.0 (42.0-62.0) | 0.3 (0.2-0.5) | 0.2 (0.1-2.5) | 3 (75.0) | 1 (25.0) | 1169.5 (318.0-5750.0) |
| *CNGB1* | AR | 3/3 | 1 : 2 | 6.0 (5.0-50.0) | 45.0 (31.0-61.0) | 46.0 (33.0-64.0) | 0.2 (0.0-0.3) | 0.2 (0.1-0.3) | 2 (66.0) | 1 (33.0) | 5170.0 (4080.0-5431.0) |
| *CRB1* | AD/AR | 2/2 | 1 : 1 | 16.5 (13.0-20.0) | 17.5 (13.0-22.0) | 21.0 (20.0-22.0) | 1.3 (0.1-2.5) | 0.9 (0.5-1.3) | 0 (0.0) | 0 (0.0) | 573.0 (327.0-819.0) |
| *HGSNAT* | AR | 1/1 | 1 : 0 | 17.0 | 39.0 | 44.0 | 1.0 | 0.7 | 1 (100.0) | 0 (0.0) | 529.0 |
| *IFT140* | AR | 1/1 | 1 : 0 | 11.0 | 17.0 | 17.0 | 0.2 | 0.2 | 0 (0.0) | 0 (0.0) | 3793.0 |
| *IMPDH1* | AD | 1/1 | 0 : 1 | 9.0 | 10.0 | 19.0 | 3.0 | 3.0 | 0 (0.0) | 0 (0.0) | 343.0 |
| *IMPG2* | AD/AR | 1/1 | 1 : 0 | 10.0 | 37.0 | 42.0 | 0.3 | 0.2 | 0 (0.0) | 0 (0.0) | 2920.0 |
| *MAK* | AR | 1/1 | 0 : 1 | 40.0 | 43.0 | 53.0 | 0.1 | 0.2 | 1 (100.0) | 1 (100.0) | 1782.0 |
| *NPHP4* | AR | 1/1 | 1 : 0 | 29.0 | 29.0 | 49.0 | 1.5 | 1.5 | 1 (100.0) | 1 (0.0) | 329.0 |
| *PCDH15* | AR/DR | 1/1 | 1 : 0 | 7.0 | 14.0 | 30.0 | 1.3 | 1.3 | 0 (0.0) | 0 (0.0) | 1200.0 |
| *PRPF31* | AD | 3/2 | 1 : 2 | 15.0 (7.0-55.0) | 15.0 (15.0-60.0) | 61.0 (40.0-72.0) | 3.0 (1.3-3.0) | 3.0 (2.5-3.0) | 1 (33.0) | 1 (33.0) | 458.0 (259.0-903.0) |
| *PRPF8* | AD | 1/1 | 0 : 1 | 13.0 | 20.0 | 21.0 | 0.2 | 0.1 | 0 (0.0) | 0 (0.0) | 2890.0 |
| *PRPH2* | AD/AR | 1/1 | 1 : 0 | 13.0 | 58.0 | 58.0 | 0.0 | 0.2 | 1 (100.0) | 0 (0.0) | 5849.0 |
| *RHO* | AD/AR | 4/4 | 3 : 1 | 30.0 (6.0-51.0) | 17.5 (26.0-51.0) | 60.0 (34.0-60.0) | 0.1 (0.0-0.7) | 0.0 (0.0-1.0) | 4 (100.0) | 1 (25.0) | 2849.0 (418.0-3827.0) |
| *RP2* | XL | 1/1 | 1 : 0 | 5.0 | 13.0 | 24 | 1.0 | 0.7 | 1 (100.0) | 0 (0.0) | 747.0 |
| *RPGR* | XL | 2/2 | 2 : 0 | 8.5 (7.0-10.0) | 19.0 (18.0-20.0) | 40.5 (30.0-51.0) | 1.4 (0.3-2.5) | 0.9 (0.3-1.5) | 2 (100.0) | 0 (0.0) | 569.0 (293.0-845.0) |
| *SNRNP200* | AD | 1/1 | 1 : 0 | 15.0 | 36.0 | 36.0 | 0.1 | 0.1 | 0 (0.0) | 0 (0.0) | 2453.0 |
| *USH1G* | AR | 1/1 | 0 : 1 | 8.0 | 40.0 | 45.0 | 2.5 | 3.0 | 1 (100.0) | 0 (0.0) | 569.0 |

Abbreviations: BCVA, best-corrected visual acuity; LogMAR, logarithm of the minimum angle of resolution; OCT, optical coherence tomography; M, male; F, female; OD, oculus dexter; OS, oculus sinister; ERM, epiretinal membrane; CME, cystoid macular edema; EZ, ellipsoid zone; AD, autosomal dominant; AR, autosomal recessive; XL, X-linked

**Supplement eTable 3.** Causative genes and variants in 80 Korean patients with retinitis pigmentosa.

| **Subject No.** | **Causative Gene** | | **NM number** | **Chromosome** | **HGVS DNA change** | **HGVS Protein change** | **Zygosity** | **Inheritance** | **Mutation type** | **ACMG criteria** | | **Population frequency**  **(Global/Korean)** |
| --- | --- | --- | --- | --- | --- | --- | --- | --- | --- | --- | --- | --- |
| ABCA4-1* | | *ABCA4* | NM_000350.3 | 1 | c.6119G>A | p.Arg2040Gln | hetero | AD/AR | missense | LP | PM1 PM2 PP3 PP5 | 0.00031/ 0.00105 |
| ABCA4-2* | | *ABCA4* | NM_000350.3 | 1 | c.880C>T | p.Gln294Ter | hetero | AD/AR | nonsense | P | PVS1 PM2 PP3 PP5 | 0.00005/0.00034 |
| ABCA4-3 | | *ABCA4* | NM_000350.3 | 1 | c.880C>T | p.Gln294Ter | hetero | AD/AR | nonsense | P | PVS1 PM2 PP3 PP5 | 0.00005/0.00034 |
| ABCA4-3 | | *ABCA4* | NM_000350.3 | 1 | c.1760+2T>G |  | hetero |  | splice donor | P^a^ | PVS1 PM2 PP3 PP5 | 0.00003/0.00039 |
| CDH23-1 | | *CDH23* | NM_022124.5 | 10 | c.1282G>A | p.Asp428Asn | hetero | AD/AR | missense | LP | PM1 PM2 BS2 | 0.00050/0.00787 |
| CDH23-1 | | *CDH23* | NM_022124.5 | 10 | c.3038G>A | p.Arg1013Gln | hetero |  | missense | VUS^a,b^ | PM1 PM2 PP3 | 0.00005/0.000 |
| CNGA1-1 | | *CNGA1* | NM_001142564.1 | 4 | c.472del | p.Leu158fs | hetero | AR | nonsense | P | PVS1 PM2 PP5 | 0.00010/0.00288 |
| CNGA1-1 | | *CNGA1* | NM_001142564.1 | 4 | c.2134C>T | p.Arg712Ter | hetero | AR | nonsense | VUS^b^ | PM2 PP3 | 0.00002/0.00052 |
| CNGA1-2 | | *CNGA1* | NM_001142564.1 | 4 | c.472del | p.Leu158fs | homo | AR | nonsense | P | PVS1 PM2 PP5 | 0.00010/0.00288 |
| CNGA1-3 | | *CNGA1* | NM_001142564.1 | 4 | c.472del | p.Leu158fs | homo | AR | nonsense | P | PVS1 PM2 PP5 | 0.00010/0.00288 |
| CNGA1-4 | | *CNGA1* | NM_001142564.1 | 4 | c.472delC | p.Leu158fs | hetero | AR | nonsense | P | PVS1 PM2 PP5 | 0.00010/0.00288 |
| CNGA1-4 | | *CNGA1* | NM_001142564.1 | 4 | c.398delG | p.Gly133fs | hetero | AR | nonsense | P | PVS1 PM2 PP5 | 0.00002/0.00164 |
| CNGB-1 | | *CNGB1* | NM_001297.4 | 16 | c.217+5G>C |  | homo | AR | intron variant | LP | PM2 PM3 PP3 PP5 | 0.00004/0.00164 |
| CNGB-2 | | *CNGB1* | NM_001297.4 | 16 | c.217+5G>C |  | homo | AR | intron variant | LP | PM2 PM3 PP3 PP5 | 0.00004/0.00164 |
| CNGB-3 | | *CNGB1* | NM_001297.4 | 16 | c.217+5G>C |  | homo | AR | intron variant | LP | PM2 PM3 PP3 PP5 | 0.00004/0.00164 |
| CRB1-1 | | *CRB1* | NM_201253.2 | 1 | c.653-2A>T |  | hetero | AD/AR |  | P^a^ | PVS1 PM2 PP3 |  |
| CRB1-1 | | *CRB1* | NM_201253.2 |  | c.1576C>T | p.Arg526Ter | hetero |  | nonsense | P | PVS1 PM2 PP3 PP5 | 0.00032/0.00079 |
| CRB1-2 | | *CRB1* | NM_201253.2 | 1 | c.1576C>T | p.Arg526Ter | hetero | AD/AR | nonsense | P | PVS1 PM2 PP3 PP5 | 0.00032/0.00079 |
| CRB1-2 | | *CRB1* | NM_201253.2 |  | c.2198A>G | p.Tyr733Cys | hetero |  | missense | VUS^b^ | PM1 PM2 | 0.00000/0.00000 |
| HGSNAT-1 | | *HGSNAT* | NM_152419.2 | 8 | c.34_54del | p.Leu12_Leu18del | hetero | AR | nonsense | VUS^a, b^ | PM2 PM4 | 0.00001/0.00000 |
| HGSNAT-1 | | *HGSNAT* | NM_152419.2 | 8 | c.1030C>T | p.Arg344Cys | hetero | AR | missense | LP | PM2 PP3 PP5 | 0.00001/0.00000 |
| IFT140-1 | | *IFT140* | NM_014714.3 | 16 | c.2551_2563del | p.Val851fs | hetero | AR | frameshift | LP^a^ | PVS1 PM2 | N/A |
| IFT140-1 | | *IFT140* | NM_014714.3 | 16 | c.1183G>A | p.Val395Met | hetero | AR | missense | VUS^a, b^ | PM1 PM2 PP3 | N/A |
| IMPDH1-1 | | *IMPDH1* | NM_000883.3 | 7 | c.947G>C | p.Arg316Pro | hetero | AD | missense | LP | PM1 PM2 PP2 PP3 PP5 | N/A |
| IMPG2-1 | | *IMPG2* | NM_016247.3 | 3 | c.1589C>A | p.Ser530Ter | hetero | AD/AR | nonsense | P | PVS1 PM2 PP5 | 0.000004/0.0000 |
| IMPG2-1 | | *IMPG2* | NM_016247.3 | 3 | c.2629A>C | p.Met877Leu | hetero |  | missense | VUS^a, b^ | PM2 BP4 | 0.00003/0.0000 |
| MAK-1 | | *MAK* | NM_001242957.2 | 6 | c.824C>A | p.Ala275Glu | hetero | AR | missense | VUS^a, b^ | PM1 PM2 PP3 | N/A |
| MAK-1 | | *MAK* | NM_001242957.2 | 6 | c.493T>A | p.Tyr165Asn | hetero | AR | missense | VUS^a, b^ | PM1 PM2 PP3 | N/A |
| NPHP4-1 | | *NPHP4* | NM_015102.4 | 1 | c.1972C>T | p.Arg658Ter | hetero | AR | nonsense | P | PVS1 PM2 PP3 PP5 | 0.00001/0.0000 |
| NPHP4-1 | | *NPHP4* | NM_015102.4 | 1 | c.453-1G>C |  | hetero | AR | splice acceptor | P | PVS1 PM2 PP3 | N/A |
| PCDH15-1 | | *PCDH15* | NM_001142763.1 | 10 | c.1799+1G>T |  | hetero | AR/DR | splice donor | P | PVS1 PM2 PP3 | N/A |
| PCDH15-1 | | *PCDH15* | NM_001142763.1 | 10 | c.1795C>T | p.Arg599Ter | hetero |  | nonsense | P^a^ | PVS1 PM2 PP3 | N/A |
| PRPF31-1* | | *PRPF31* | NM_015629.3 | 19 | c.1120C>T | p.Gln374Ter | hetero | AD | nonsense | P | PVS1 PM2 PP3 PP5 | N/A |
| PRPF31-2 | | *PRPF31* | NM_015629.3 | 19 | c.320T>C | p.Leu107Pro | hetero | AD | missense | LP | PVS1 PM2 PP3 PP5 | N/A |
| PRPF31-3* | | *PRPF31* | NM_015629.3 | 19 | c.1120C>T | p.Gln374Ter | hetero | AD | nonsense | P | PM1 PM2 PP3 PP5 | N/A |
| PRPF8-1* | | *PRPF8* | NM_006445.3 | 17 | c.6902C>T | p.Pro2301Leu | hetero | AD | missense | VUS^a^ | PM1 PM2 PP3 | N/A |
| PRPH2-1 | | *PRPH2* | NM_000322.4 | 6 | c.478C>T | p.Gln160Ter | hetero | AD/AR | nonsense | P^a^ | PVS1 PM2 PP3 | N/A |
| RHO-1 | | *RHO* | NM_000539.3 | 3 | c.36del | p.Phe13fs | hetero | AD/AR | frameshift | P | PVS1 PM2 PP5 | 0.000007/0.00026 |
| RHO-1 | | *RHO* | NM_000539.3 | 3 | c.310G>A | p.Val104Ile | hetero |  | missense | VUS ^b^ | PM1 PM2 | 0.00020/0.00105 |
| RHO-2 | | *RHO* | NM_000539.3 | 3 | c.36del | p.Phe13fs | hetero | AD/AR | frameshift | P | PVS1 PM2 PP5 | 0.000007/0.00026 |
| RHO-3 | | *RHO* | NM_000539.3 | 3 | c.50C>T | p.Thr17Met | hetero | AD/AR | missense | LP | PM1 PM2 PP3 PP5 | 0.00003/0.00000 |
| RHO-4 | | *RHO* | NM_000539.3 | 3 | c.965_966delinsAA | p.Cys322Ter | hetero | AD/AR | nonsense | LP^a^ | PVS1 PM2 | N/A |
| RHO-4 | | *RHO* | NM_000539.3 | 3 | c.994G>C | p.Glu332Gln | hetero |  | missense | VUS^a, b^ | PM2 | N/A |
| RP2-1* | | *RP2* | NM_006915.2 | X | c.353G>A | p.Arg118His | hemi | XL | missense | LP | PM1 PM2 PP3 PP5 | N/A |
| SNRNP200-1 | | *SNRNP200* | NM_014014.4 | 2 | c.2041C>T | p.Arg681Cys | hetero | AD | missense | LP | PM1 PM2 PP3 PP5 | 0.000004/0.00000 |
| RPGR-1 | | *RPGR* | NM_001034853.1 | X | c.2405_2406del | p.Pro802fs | hemi | XL | frameshift | P | PVS1 PM2 PP5 | N/A |
| RPGR-2 | | *RPGR* | NM_001034853.1 | X | c.2032G>T | p.Glu678Ter | hemi | XL | nonsense | LP^a^ | PVS1 PM2 | N/A |
| USH1G-1* | | *USH1G* | NM_173477.4 | 17 | c.164+5G>A |  | homo | AR |  | VUS^a^ | PM2 PP3 | N/A |

Abbreviations: ACMG, American College of Medical Genetics and Genomics; HGVS, human genome variation society; P, pathogenic variant; LP, likely pathogenic variant; VUS, variant unknown significance; AR, autosomal recessive; AD, autosomal dominant; XL, x-linked; hetero, heterozygote; homo, homozygote; hemi, hemizygote

*; patients who underwent segregation test, a; novel variants, b; variants confirmed by definite phenotype of retinitis pigmentosa
